# The dissociative subtype of posttraumatic stress disorder is associated with subcortical white matter network alterations

**DOI:** 10.1007/s11682-020-00274-x

**Published:** 2020-04-27

**Authors:** Anika Sierk, Antje Manthey, Eva-Lotta Brakemeier, Henrik Walter, Judith K. Daniels

**Affiliations:** 1 Charité - Universitätsmedizin Berlin, corporate member of Freie Universität Berlin, Humboldt-Universität zu Berlin, and Berlin Institute of Health, Berlin, Germany, Berlin, Germany; 2 Institute of Cognitive Neuroscience, University College London, London, UK; 3 Department of Psychology & Marburg Center for Mind, Brain and Behavior (MCMBB), Philipps-Universität Marburg, Marburg, Germany; 4 Department of Clinical Psychology, University of Groningen, Groningen, Netherlands; 5 Psychologische Hochschule Berlin, Berlin, Germany

**Keywords:** Network-based statistics, Diffusion MRI, Tractography, PTSD, Dissociation, Trauma

## Abstract

Posttraumatic stress disorder (PTSD) is characterized by intrusions, avoidance, and hyperarousal while patients of the dissociative subtype (PTSD-D) experience additional dissociative symptoms. A neurobiological model proposes hyper-inhibition of limbic structures mediated by prefrontal cortices to underlie dissociation in PTSD. Here, we tested whether functional alterations in fronto-limbic circuits are underpinned by white matter network abnormalities on a network level. 23 women with PTSD-D and 19 women with classic PTSD participated. We employed deterministic diffusion tractography and graph theoretical analyses. Mean fractional anisotropy (FA) was chosen as a network weight and group differences assessed using network-based statistics. No significant white matter network alterations comprising both frontal and limbic structures in PTSD-D relative to classic PTSD were found. A subsequent whole brain exploratory analysis revealed relative FA alterations in PTSD-D in two subcortical networks, comprising connections between the left amygdala, hippocampus, and thalamus as well as links between the left ventral diencephalon, putamen, and pallidum, respectively. Dissociative symptom severity in the PTSD-D group correlated with FA values within both networks. Our findings suggest fronto-limbic inhibition in PTSD-D may present a dynamic neural process, which is not hard-wired via white matter tracts. Our exploratory results point towards altered fiber tract communication in a limbic-thalamic circuit, which may underlie (a) an initial strong emotional reaction to trauma reminders before conscious regulatory processes are enabled and (b) deficits in early sensory processing. In addition, aberrant structural connectivity in low-level motor regions may present neural correlates for dissociation as a passive threat-response.

**Electronic supplementary material:**

The online version of this article (10.1007/s11682-020-00274-x) contains supplementary material, which is available to authorized users.

## Introduction

Posttraumatic stress disorder (PTSD) is one of the commonest trauma-related disorders with a life time prevalence of 6.8% in the general population (Kessler et al. 2005). PTSD is characterized by intrusions, avoidance, and hyperarousal, with some patients experiencing additional dissociative symptoms such as depersonalization and derealization (American Psychiatric Association 2013). Over the past years, several empirical studies indicated that pronounced dissociative symptomatology might not be represented dimensionally in PTSD but can be attributed to a distinct subgroup of patients. The dissociative subtype of PTSD, abbreviated with “PTSD-D” in the present work, was recently included in the DSM-5 (American Psychiatric Association 2013). In support of this novel sub-distinction, different research groups conducted latent class analyses, suggesting 12%–29.9% of patients to belong to this subtype (Armour and Hansen 2015; Steuwe et al. 2012; Tsai et al. 2015; Waelde et al. 2005; Wolf et al. 2012) with higher prevalence rates in women (Wolf et al. 2012) and in participants having experienced childhood sexual abuse (Steuwe et al. 2012; Wolf et al. 2012), independent of gender (Yiaslas et al. 2014). Most of these studies found that patients with PTSD-D displayed higher symptom severity mediated by higher intrusive symptomatology.

It has been suggested that dissociative states in PTSD are associated with distinct physiological and neural activation patterns (Lanius et al. 2010). Psychophysiological studies are not conclusive yet, but tend to indicate that non-dissociative patients display heightened heart rate during trauma-exposure (for review see Bedi and Arora 2007), while dissociative patients display unaltered or slightly lower heart rate during acute dissociation (Griffin et al. 1997; Lanius et al. 2002). Using functional neuroimaging (fMRI), the working group around Lanius studied patients during acute dissociation and found relatively reduced blood flow in structures crucial for emotion processing (amygdala and insula; Daniels et al. 2012; Mickleborough et al. 2011) and heightened blood flow in regions associated with cognitive control of affective responses (medial prefrontal cortex and rostral anterior cingulate cortex; Daniels et al. 2012; Hopper et al. 2007). The authors propose that during dissociation, prefrontal cortices overregulate limbic structures, while during intrusive re-experiencing, deficient prefrontal inhibition leads to limbic hyperactivation (cf. Lanius et al. 2010; also see Liberzon and Garfinkel 2009). These opposing neuronal patterns of emotional over- and underegulation co-exist in patients with PTSD-D per definition, suggesting dissociation to be either underpinned by dynamic neural processes or to be hardwired via distinct fronto-limbic pathways.

The same group recently suggested that most of their functional connectivity findings are in line with this model (see Fig. 3, page 118 of Lanius et al. 2018). These findings can be summarized as indicating that the dissociative subtype of PTSD is characterized by greater functional connectivity between the amygdala and several brain regions involved in emotion regulation and interoceptive awareness. However, most of these studies employed a seed-based analysis, i.e. testing an assumed relationship with a very narrowly defined brain region, and have not been replicated yet. To our knowledge, no whole brain network-based analyses are available at the moment, which use an assumption-free, explorative approach.

Two studies have reported correlations between brain morphology and dissociative symptom severity in PTSD. Daniels et al. (2016) found increased volume of the right precentral and fusiform gyri and reduced volume in right inferior temporal gyrus in patients with PTSD-D compared to patients with classic PTSD. Dissociative symptoms severity was positively associated with grey matter volume of the right middle frontal gyrus. Nardo et al. (2013) found positive correlations between trait dissociation and grey matter volume in frontal, temporal, and inferior parietal cortices. These findings indicate that emotional overregulation in PTSD-D may be underpinned by differences in grey matter brain anatomy, which could either represent pre-morbid biological risk factors for dissociative responses or adaptations to their development. Yet, these structural aberrations only referred to locally distinct areas and no interaction with brain circuits can be inferred from these studies. It thus remains unclear whether the observed symptomatology is further underpinned by structural alterations of the white matter in PTSD-D. Various studies have reported white matter alterations in PTSD (Daniels et al. 2013; Dennis et al. 2019; Siehl et al. 2018). Yet, to our knowledge, no study to date has differentiated between the subtypes while again – only local aberrations have been investigated for the exception of one recent study, which reported differences in white matter network organization in PTSD compared to trauma-exposed controls (Suo et al. 2019).

Investigating the white matter tract communication on a network level presents a promising approach. Diffusion weighted imaging (DWI) allows to image the human brain connectome non-invasively (Derek K. Jones and Leemans 2011; J.-D. Tournier et al. 2011), while the combined usage of tractography and graph theory enables the analysis of structural connectivity on a network level (Bullmore and Sporns 2009; Fornito et al. 2013; Griffa et al. 2013; Zalesky et al. 2010). In the present study, we apply graph theoretical analyses on data of diffusion MRI tractography to identify sub-networks with distinct structural connectivity between PTSD-D patients and patients with classic PTSD. Firstly, we test whether patients with PTSD-D and classic PTSD differ with regard to their structural connectivity in fronto-limbic circuits as hypothesized based on the model of limbic overregulation. However, to our knowledge, no study to date has analyzed structural connectivity, that is white matter anatomy, in PTSD-D. Therefore, we secondly carry out an exploratory whole-brain analysis aimed at theory building.

## Methods

### Participants

The study sample consisted of participants who had experienced childhood trauma (interpersonal abuse) and presented chronic PTSD, that is, they have suffered from PTSD for at least three months. Diffusion imaging scans were acquired in 45 women with PTSD (mean age 40.0 ± 9.8 ys, see Table 2 for further demographics). One participant could not be clearly categorized into either the classic PTSD or the PTSD-D group (cf. section 2.3) and thus, was excluded from the present analysis. Furthermore, two patients of the PTSD-D group had to be excluded due to incidental findings by a neuro-radiologist, leaving in total 23 women in the PTSD-D group and 19 women in the classic PTSD group.

Study participants were recruited via public advertisements, in collaboration with licensed psychotherapists and psychiatrists, or through mental health in- and outpatient clinics. Participants were eligible for the study if they met the following criteria: (1) between 20 and 60 years old, (2) proficient in German, (3) MRI compatible, (4) no neurological disorder, (5) no history of head injury, (6) no substance dependency, (7) no intake of benzodiazepines or anticonvulsants (subjects taking only antidepressant medication were included), and (8) PTSD as their primary disorder. If presented as the secondary diagnosis (i.e. symptoms were not as severe in intensity as the PTSD symptomatology), we allowed comorbid depressive and anxiety disorders, eating disorders, borderline personality disorder, and substance abuse disorders in order to ensure ecological validity. All other Axis-I disorders were excluded, with special attention given to the exclusion of comorbid dissociative disorders to disambiguate the diagnostic status. Written informed consent was obtained from the participants prior to participation and approval of the study was granted by the ethics board at the department of medicine at the University of Magdeburg and the ethical committee of the Berlin Psychological University.

### Procedure

#### Clinical diagnostics

All participants were pre-screened on the telephone regarding MRI incompatibilities, head injuries, medication, and current psychological as well as neurological disorders. Subsequently, we sent out a questionnaire package including German versions of the Essen Trauma Inventory (Tagay et al. 2006), the Dissociative Experiences Scale (DES; Spitzer et al. 2003), and the PTSD Checklist for DSM-IV (PCL; Teegen 1997) to screen for trauma exposure and PTSD symptoms, respectively. Eligible participants were invited for a diagnostic assessment by a licensed clinical psychologist. German versions of four standardised clinical interviews were implemented. (1) The Clinician-administered PTSD Scale (CAPS-IV; Schnyder and Moergeli 2002) was used to establish the PTSD diagnosis, and (2) the Structured Clinical Interview for DSM-IV (Wittchen et al. 1997) was implemented for the diagnosis of Axis-I disorders. To exclude subjects with dissociative disorders or primary borderline personality disorder, (3) the Structured Clinical Interview for DSM-IV Dissociative Disorders (SCID-D; Gast et al. 2000) and (4) the respective section of the Structured Clinical Interview for DSM-IV axis II (Fydrich et al. 1997) were employed.

#### Questionnaires and tasks

All participants completed several self-report questionnaires. To assess trait and state dissociation, German versions of the 30-item and 22-item Cambridge Depersonalization Scale (CDS-30; CDS-state; Michal et al. 2004) were implemented, respectively. Further questionnaires to characterize the dissociative experience were the Multiscale Dissociation Inventory (MDI; Brière 2002; authorized German translation by J. Daniels [unpublished, University of Groningen, The Netherlands]), the Peritraumatic Dissociative Experiences Questionnaire (PDEQ; Marmar et al. 1994; authorized German translation by A. Maercker [unpublished, TU Dresden, Germany]), and the Somatoform Dissociation Questionnaire (SDQ-20; Mueller-Pfeiffer et al. 2010). For further sample characterization, we employed the Beck Depression Inventory (BDI-II, Hautzinger et al. 2006), the Emotion Regulation Questionnaire (ERQ; Abler and Kessler 2009), the State-Trait Anxiety Inventory (STAI-T; Laux and Spielberger 2001), and the Childhood Trauma Questionnaire (CTQ; Wingenfeld et al. 2010). In addition, information processing speed and executive functions were assessed using the Trail Making Test versions A and B (TMT; Stanczak et al. 1998), respectively.

### Subtype allocation

The classification of participants into either the classic PTSD or the PTSD-D group was based on five diagnostic instruments: DES, CDS-30, CDS-state, CAPS, and SKID-D. Pre-defined cut-offs for each questionnaire indicated whether dissociative symptoms were prevalent or not. If patients scored above the cut-off in at least three of these five instruments, they were diagnosed with PTSD-D. Accordingly, if they scored below the cut-offs in at least three questionnaires, participants were allocated to the classic PTSD group. We specified the following cut-offs: (1) ≥ 20 in the DES, (2) ≥ 20 in the CDS-30 (only frequency; cf. Spitzer et al., 2015), (3) CDS-state ≥15, (4) ≥ 4 in two questions assessing depersonalization and derealization in the CAPS, (5) ≥ 4 in the two SKID-D sections measuring depersonalization and derealization, respectively.

Two participants could not be clearly classified into the PTSD or PTSD-D. One participant displayed high dissociative scores on the two self-report questionnaires but low scores regarding dissociation on the clinical interviews. We decided to exclude this participant from the analysis (cf. section 2.1), due to the strong incongruence between self- and external assessment. Another participant scored clearly below the cut-off in the CDS-30 and the SCID-D, but just above the cut-offs in all remaining three questionnaires. We decided to allocate this participant to the classic PTSD group, because of the relative congruency between self- and external assessment.

### MRI acquisition

Diffusion images and T1-weighted images were acquired on a 3 T Siemens Tim Trio scanner (Siemens, Erlangen, Germany) equipped with a 12-channel head coil. Diffusion imaging was performed with a single-shot echo-planar imaging sequence using the following parameters: TR = 7500 ms, TE = 86 ms, 61 slices, voxel size = 2.3 × 2.3 × 2.3mm^3^, slice thickness = 2.3 mm, FOV = 220x220mm^2^, 64 diffusion directions, b value = 1000s/mm^2^, phase-encoding direction = anterior to posterior. Structural T1-weighted images were obtained with a magnetization-prepared rapid acquisition with gradient echo sequence (TR = 1.9 ms, TE = 2.52 ms, inversion time = 900 ms, flip angle = 9°, FoV = 256x256mm^2^, 192 slices, 1 mm isotropic voxel sizes, 50% distancing factor).

### Preprocessing

**Fig. 1:**
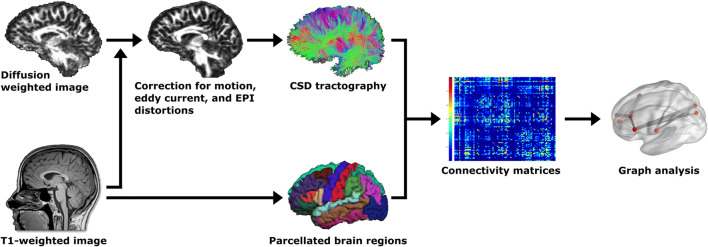
Flowchart of the preprocessing pipeline, which was performed using *FreeSurfer* (https://surfer.nmr.mgh.harvard.edu) and *ExploreDTI* (http://www.exploredti.com). EPI = echo-planar imaging, CSD = constrained spherical deconvolution

The preprocessing pipeline for the structural network analysis is provided as a flow chart in Fig. 1 with arrows indicating the order of steps. The T1-weighted MRI scans were processed with the automated image-processing software *FreeSurfer v6.0* (https://surfer.nmr.mgh.harvard.edu/). Important processing steps include skull stripping, segmentation of subcortical white matter and deep gray matter volumetric structures, definition of the grey and white matter boundaries, and parcellation of the cerebral cortex (Fischl and Dale 2000). We used the default settings implemented in *FreeSurfer*. Each output was visually inspected for quality control.

The diffusion data was preprocessed using the default parameter settings in *ExploreDTI*, version 4.8.6 (http://www.exploredti.com; A Leemans et al. 2009). As part of preprocessing, images were corrected for subject motion using the ‘Rekindle’ method (Chantal MW Tax et al. 2015), eddy current induced geometric distortions (A. Leemans and Jones 2009), and EPI distortions (Irfanoglu et al. 2012). Constrained spherical deconvolution whole brain tractography was performed (Jeurissen et al. 2011; C. M. Tax et al. 2014), which results in one streamline file per subject. Each processing step was visually inspected for quality insurance as well as valid co-registration checked by overlaying the respective images for each subject.

### Connectivity matrices

To construct structural connectivity matrices, we used 87 predefined anatomical regions of interests (ROIs) derived from *FreeSurfer*. ROIs comprised all cortical regions from the Desikan Killiany atlas (35 areas) as well as the bilateral subcortical structures amygdala, hippocampus, caudate, putamen, pallidum, accumbens-area, thalamus, ventral diencephalon (DC), and the brain-stem. The ventral DC refers to a miscellaneous area, which comprises smaller nuclei and structures inferior to the thalamus (hypothalamus, red nuclei, medial and lateral geniculate nuclei, mammillary body, subthalamic nuclei, and substantia nigra as well as surrounding white matter). These ROIs were combined with the information in the streamline files available for each subject. Specifically, for all possible ROI-pairs, the number of streamlines between two ROIs was counted given they passed through both ROIs (i.e., the ‘PASS’ setting in *ExploreDTI* was used). The resulting number of streamlines were converted to symmetrical 87 × 87 matrices, which were stored for subsequent network analysis.

Due to the deterministic tracking algorithm used, not all possible fiber tracts can be reconstructed in all subjects (Jeurissen et al. 2017; Maier-Hein et al. 2017). As this may vary between groups, we aimed to only include links in the network analyses for which fibers were tracked successfully in all participants. However, this restriction resulted in only 190 links to be entered into the analysis and we considered this procedure to be too conservative, potentially inflating false negative results. Hence, we chose to threshold the connectivity matrices by a minimum number of streamlines (maximum number of tracts in each subject * .001), which still curbs the effect of spurious streamlines (cf. Rubinov and Sporns 2010), but allowed us to include all possible 87 × 87 links into the analysis.

### Statistics of network analyses

The second-level network analyses (i.e. network-based statistics, and partial correlational analyses on a network level) were performed on the streamline matrices using the software GraphVar 1.3 (http://www.nitrc.org/projects/graphvar/; Kruschwitz et al. 2015), a toolbox running in MATLAB R2016b (https://mathworks.com). The streamlines between each pair of ROIs were weighted, that is, the streamlines between each pair of ROIs were used as a mask, within which we computed mean fractional anisotropy (FA). We chose FA as an edge weight, because it is sensitive to microstructural changes of white matter and thus, may provide indication for pathologic changes or altered structural connectivity (Hasan et al. 2004).

### Network-based statistics

We used network-based statistics (NBS) to test for group differences in structural connectivity between the PTSD-D and classic PTSD group. NBS is a nonparametric statistical method developed by Zalesky et al. (2010), which can be used to identify graph components within a network that are associated with an external variable, in our case group membership, while controlling the family wise error rate (FWER). Within NBS, statistical thresholding is performed in two steps: First, at every connection within a network, the hypothesis of interest is tested independently using so called initial link-thresholds. Resulting supra-threshold links may eventually form graph components. Whether any of these graph components are significant at the network level is determined by their size, which is compared to the occurrence of differently sized graph components derived from random data (i.e. by performing FWE-correction).

According to this procedure, we performed a series of t-tests to identify links between pre-defined ROIs (see above) for which the PTSD-D and classic PTSD group displayed significant differences in their structural connectivity (i.e. FA). We applied two initial link thresholds (lt) of *p*_*lt*_ = .005 and *p*_*lt*_ = .001. Following procedures in our previous paper (Sierk et al. 2018), we chose more than one initial link threshold to obtain information regarding the nature of any observed group difference. Effects evident only at liberal thresholds (e.g. *p*_*lt*_ < .05) are rather subtle and topologically extended, whereas effects found at conservative thresholds (e.g. *p*_*lt*_ < .001) are likely to disclose strong focal differences between groups (cf. Zalesky et al. 2010).

The significance of an identified graph component (i.e., a sub-network) was determined by generating a corresponding null-model distribution, employing 10,000 permutations. We considered an identified sub-network statistically significant with an FWER-corrected *p* value of *p*_*FWER*_ < .05. Note that multiple comparison correction is performed on a network level and thus, only the networks as a whole is considered significant and can only be interpreted as such (cf. Zalesky et al. 2010).

### Group comparison: PTSD vs. PTSD-D

We performed NBS to test for group differences in structural connectivity between patients with PTSD and PTSD-D using a (1) hypothesis-driven approach followed by (2) an exploratory approach used for potential theory-building.

To test for significant group differences in structural connectivity between brain regions implicated in the proposed model of fronto-limbic dysbalance, solely limbic and prefrontal regions were selected. In regard to limbic structures, we selected regions from the FreeSurfer parcellation (Desikan Killany atlas), which are proposed to belong to the limbic system (cf. Isaacson 2013) and which have been reported to be undermodulated in PTSD-D (Lanius et al. 2010). Regarding frontal structures, we selected all parcellated regions within the frontal lobe (Lanius et al. 2010). This resulted in 8 limbic and 10 frontal ROIs, each tested bilaterally. The respective regions are listed in Table 1. Results were considered relevant if a sub-network was detected, which included both at least one frontal and one limbic region.

**Table 1:** Bilateral frontal and limbic structures that were entered in the first analysis, testing for group differences regarding the model of fronto-limbic dysbalance

Limbic structures	Frontal structures
Hippocampus	Caudal middle frontal gyrus
Amygdala	Rostral middle frontal gyrus
Accumbens area	Lateral orbitofrontal cortex
Ventral diencephalon	Medial orbitofrontal cortex
Insula	Pars opercularis
Caudal anterior cingulate cortex	Pars orbitalis
Rostral anterior cingulate cortex	Pars triangularis
Posterior cingulate cortex	Parahippocampal gyrus
	Superior frontal gyrus
	Frontal pole

For the exploratory whole-brain analysis of network-level FA differences between the PTSD-D and the classic PTSD group, we included all possible links (87 × 87 ROIs) into the analysis.

### Correlational analyses

To test whether any identified group differences are related to dissociative symptomatology, we subjected the connectivity matrices of the PTSD-D and the classic PTSD group to a partial correlation analysis with dissociative symptom severity, as measured by the CDS-30 (controlled for age). The CDS-30 was used as it specifically assesses depersonalization and derealization and was employed to the same end in our previous study on connectivity alterations in patients with a dissociative disorder (Sierk et al. 2018). To obtain the respective sets of supra-threshold links, we employed partial correlations for mass-univariate testing in each cell of the connectivity matrix. As described in the previous section, significance of any identified graph components was tested by applying permutation testing using 10,000 random permutations of CDS-30 scores. Pearson correlations were computed across the PTSD-D and the classic PTSD group, separately.

Age was included as a covariate in all network analyses.

## Results

### Demographics

Group differences regarding demographic information are listed in Table 2. Patients with PTSD-D did not differ from the classic PTSD group regarding age (*t(40)* = 0.12, *p* = .908), level of education (Mann–Whitney *U* = 192.00, *p* = .423), information processing speed (TMT-A; *t(40)* = 0.74, *p* = .461), and executive functions (TMT-B; *t(40)* = 0.57, *p* = .570). As shown in Table 2, no group differences were detected regarding depressive symptoms (i.e. BDI-II scores), trait anxiety (i.e. STAI-T scores), emotion regulation (i.e. ERQ scores), and childhood trauma experiences (i.e. CTQ scores). As expected, PTSD-D patients scored significantly higher than patients with classic PTSD on measures of trait dissociation (DES, *t(40)* = −3.21, *p =* .003), current dissociation (CDS-30, *t(37)* = −7.11, *p* < .001; MDI, *t(37)* = −4.11, *p* < .001), state dissociation (CDS-state, *t(39)* = −4.30, *p* < .001), somatoform dissociation (SDQ-20, *t(37)* = −3.42, *p* = .002), and peritraumatic dissociation (PDEQ, *t(37)* = −3.58, *p* < .001). There was a non-significant trend pointing towards higher PTSD symptom severity, as measured by the CAPS, in the PTSD-D compared to the classic PTSD group (*t(40)* = −1.80, *p* = .079). The questionnaires measuring dissociation correlated significantly with each other as well as with BDI and STAIT scores (see Online Resource 2).

**Table 2:** Group differences regarding demographics and clinical measures

	Classic PTSD		PTSD-D		Statistics (two-tailed t-test)		
Variable	n	Mean (SD)	n	Mean (SD)	*t* score	*df*	*p* value
Age	19	40.32 (9.44)	23	39.96 (10.38)	.12	40	.908
**CDS-30**	**17**	**11.82 (8.86)**	**22**	**42.23 (17.36)**	**−7.11**	**37**	**<.001**
**CDS-state**	**19**	**105.26 (177.93)**	**22**	**504.09 (390.37)**	**−4.30**	**39**	**<.001**
**DES**	**19**	**21.29 (14.35)**	**23**	**35.70 (14.60)**	**−3.21**	**40**	**.003**
**MDI**	**17**	**50.18 (18.12)**	**22**	**76.27 (20.79)**	**−4.11**	**37**	**<.001**
**PDEQ**	**17**	**17.65 (10.07)**	**22**	**27.55 (7.20)**	**−3.58**	**37**	**.001**
**SDQ-20**	**17**	**28.35 (8.37)**	**22**	**40.41 (12.50)**	**−3.42**	**37**	**.002**
BDI-II	17	23.06 (13.95)	23	22.52 (13.51)	.12	38	.903
CAPS	19	64.63 (11.70)	23	71.96 (14.19)	−1.80	40	.079
CTQ total	17	83.65 (12.74)	23	86.83 (12.71)	−.78	38	.440
CTQ-PA	17	11.29 (5.97)	23	11.65 (5.34)	−.20	38	.843
CTQ-PN	17	11.41 (5.20)	23	12.43 (4.87)	−.64	38	.527
CTQ-EA	17	15.71 (3.89)	23	16.43 (4.24)	−.56	38	.581
CTQ-EN	17	18.53 (4.05)	23	19.22 (5.56)	−.43	38	.668
CTQ-SA	17	13.47 (6.78)	23	15.35 (7.54)	−.81	38	.422
ERQ-R	17	24.65 (7.30)	22	24.14 (8.10)	.20	37	.840
ERQ-S	17	18.53 (6.19)	22	15.14 (4.83)	1.93	37	.062
PCL	19	36.32 (7.19)	23	40.22 (6.05)	−1.91	40	.063
STAI-T	17	54.76 (10.30)	23	58.7 (10.62)	−1.17	38	.249
TMT-A	19	26.67 (9.60)	23	24.77 (6.90)	.74	40	.461
TMT-B	19	66.52 (35.58)	23	61.31 (22.97)	.57	40	.570

BDI=Beck Depression Inventory; CAPS=Clinician-Administered PTSD Scale; CDS=Cambridge Depersonalization Scale; CTQ = Childhood Trauma Questionnaire; DES = Dissociative Experiences Scale; df = degrees of freedom; EA = emotional abuse; EN = emotional neglect; ERQ-R = Emotion Regulation Questionnaire Reappraisal; ERQ-S = Emotion Regulation Questionnaire Suppression; MDI = Multiscale Dissociation Inventory; PA = physical abuse; PDEQ = Peritraumatic Dissociative Experiences Questionnaire; PN = physical neglect; PTSD = Posttraumatic stress disorder, PTSD-D = dissociative subtype of PTSD; SA = sexual abuse; SD = standard deviation; SDQ = Somatoform Dissociation Questionnaire; STAI-T = State-Trait Anxiety Scale, trait version; TMT = Trail Making Test (Part A and B)

Regarding comorbidity and medication, 19 PTSD-D patients and 13 classic PTSD patients displayed comorbid disorders (cf. Table 3 for details) and two patients in the PTSD-D used antidepressant medication (Valdoxan and Escitalopram, respectively).

**Table 3:** Current (and where available also past) comorbid disorders among study participants, listed separately for the two groups classic PTSD (*n* = 19) and PTSD-D (*n* = 23). All comorbid disorders present the secondary diagnosis to PTSD

		Classic PTSD *n* (past included)	PTSD-D *n* (past included)
Anxiety disorders	Generalized anxiety disorder	2	3
	Social anxiety disorder	7	11
	Specific phobia	3	1
	Panic disorder	3	7
	Agoraphobia without history of panic disorder	2	2
	Obsessive-compulsive disorder	0	3
	Total anxiety disorders	13	16
Mood disorders	Major depressive disorder, single episode	1 (3)	1 (1)
	Major depressive disorder	2 (6)	4 (12)
	Dysthymia	0 (0)	1 (0)
	Total mood disorders	3 (7)	5 (13)
Other	Substance use disorder	0 (4)	1 (4)
	Borderline personality disorder	2	6
	Eating disorder	0	4
	Somatoform disorder	0	1
Total comorbidity		13 (15)	19 (20)

### Network-based statistics

#### Group comparisons

No significant group differences emerged on a network level in fronto-limbic circuits, i.e. between any of the pre-defined frontal and limbic structures (cf. Table 1), at neither initial-link threshold (*p*_*lt*_ < .005 or *p*_*lt*_ < .001).

**Fig. 2:**
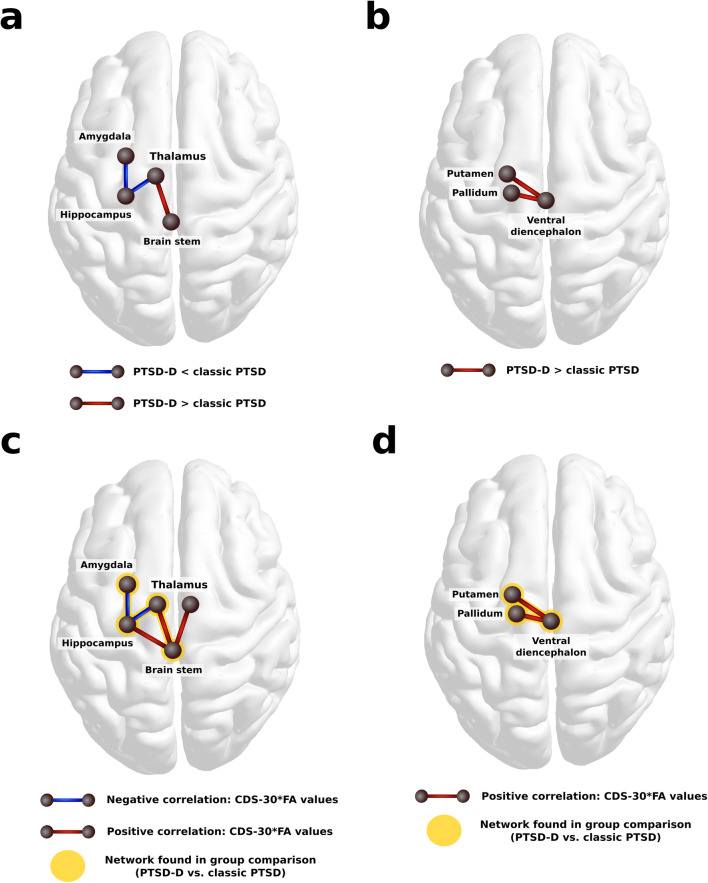
Visualization of the results found in the group comparison (a and b) and in the partial correlational analyses (c and d), both controlled for age. In the group comparison, two sub-networks were identified, in which patients with PTSD-D displayed altered FA values compared to patients with classic PTSD. a: Sub-network, in which patients with PTSD-D displayed relatively lower FA (blue connections) between the left amygdala, left hippocampus, left thalamus and higher FA (red connection) between the left thalamus and the brain stem (*p*_*FWER*_ = .026). b: Sub-network, in which PTSD-D Patients displayed higher FA between left pallidum, left ventral DC, and left putamen compared to the classic PTSD group (*p*_*FWER*_ = .027). c: Visualization of first sub-network for which FA values correlated with dissociative symptom severity (CDS-30 scores) in the PTSD-D group only (*p*_*FWER*_ = .027). d: Visualization of second subnetwork for which FA values correlated with dissociative symptom severity in patients with PTSD-D (*p*_*FWER*_ = .029). Blue connections indicate negative and red connections represent positive correlation between FA and CDS-30 scores. Yellow highlights underneath nodes and edges demonstrate the overlap between the two networks found in the partial correlation analysis and the networks identified in the group contrast. CDS=Cambridge Depersonalization Scale; DC = diencephalon; FA = fractional anisotropy; FWER = family wise error rate; PTSD = posttraumatic stress disorder; PTSD-D = dissociative subtype of posttraumatic stress disorder

In the exploratory whole-brain analysis, two sub-networks were identified at an initial-link threshold of *p*_*lt*_ < .005, for which patients with PTSD-D displayed altered FA compared to patients with classic PTSD (*p*_*FWER*_ = .026). The first network comprised four subcortical regions interconnected via three edges. Within this sub-network, the PTSD-D group showed relatively lower FA between the left amygdala and the left hippocampus as well as between the left hippocampus and left thalamus and higher FA values between the left thalamus and the brain stem (cf. Figure 2a). The second network comprised three nodes and two links between the left ventral DC and the left putamen, and left pallidum, respectively (cf. Figure 2b). Within this sub-network, patients with PTSD-D displayed higher FA values compared to patients with classic PTSD. We verified that, for all participants, tracts have been reconstructed successfully for the identified links. In this exploratory analysis, no group differences were detected at an initial-link threshold of *p*_*lt*_ < .001.

#### Partial correlation analyses

For three patients, no questionnaire data on current dissociation severity were available, leaving 17 in the classic PTSD group and 22 in the PTSD-D group for the respective partial correlation analyses (controlled for age). Applying an initial-link threshold of *p*_*lt*_ < .005, the findings held within the PTSD-D group when white matter alterations were correlated with a continuous measure of depersonalization/derealization symptoms (CDS-30). A 5-node network (*p*_*FWER*_ = .027; cf. Figure 2c) and a 3-node-network (*p*_*FWER*_ = .029; cf. Figure 2d) were detected, which overlapped with the sub-networks identified in the exploratory group comparison. However, it should be noted that this measure was one of the five instruments used for group classification. All details for this partial correlation are provided in Online Resource 1.

#### Post hoc analyses

Considering the high comorbidity with depressive and anxiety disorders in our sample as well as significant inter-correlations between questionnaires assessing anxiety, depression, and dissociation (cf. Online Resource 2), further verification was warranted to confirm whether associations between FA values and CDS-30 scores in the PTSD-D group were specifically driven by dissociation severity. Thus, we performed additional partial correlation analyses (controlling for age) between anxiety (STAI-T scores), depressive symptoms (BDI-II scores) and FA values on a network level in the PTSD-D group (initial-link threshold of *p*_*lt*_ < .005). The results are provided in table format in the Online Resources 3 and 4, respectively. The identified sub-networks did not overlap with any links found in the exploratory group contrast. Thus, employing these results as exclusive masks, we determined that FA values of the two subcortical networks correlated solely with dissociative symptom severity.

In addition, the present sample comprised more patients in the PTSD-D group who displayed secondary comorbid borderline personality disorder (*n* = 6) in comparison to patients in the classic PTSD group (*n* = 2). Therefore, we excluded patients with comorbid borderline personality disorder and reran the group analysis. The results only minimally diverted from the original group contrast, indicating that group differences cannot be explained by differences in comorbidity (cf. Online Resource 5).

## Discussion

This is the first study to have investigated differences in structural connectivity between female patients with a history of childhood trauma suffering from the dissociative subtype of PTSD (PTSD-D) versus classic PTSD. The a priori hypothesized connectivity differences involving fronto-limbic structures were not confirmed. Subsequent exploratory analyses revealed subcortical white matter alterations in two sub-networks in patients with PTSD-D relative to patients with classic PTSD, which also showed a significant correlation with dissociation severity in patients with PTSD-D, but not classic PTSD.

The null-finding regarding group differences in structural connectivity in fronto-limbic circuits suggests either that fronto-limbic inhibition in PTSD-D presents a dynamic neural process, which is not hard-wired via white matter tracts, or that frontal structures play a less central role than previously assumed. Most support for the fronto-limbic dysbalance model of PTSD-D to date has emerged from functional activation as well as functional connectivity studies (for review see Lanius et al. 2010; Nicholson et al. 2017), which both measure changes in blood flow and are methods geared to capture dynamic activity patterns in the brain. Moreover, the co-existent emotional over- and under-modulation in individuals with PTSD-D suggests dynamic response patterns that are mediated by metabolic changes and might not require underlying structural alterations. However, our null-finding also indicates that symptoms of depersonalization and derealization in PTSD might differ neurobiologically from the same symptoms in depersonalization/derealization disorders, for which we recently reported white matter network alterations in fronto-limbic as well as temporal structures (Sierk et al. 2018).

Our exploratory results may instead indicate that phenomenological differences in PTSD-D relative to classic PTSD are associated with altered white matter connectivity in subcortical circuits. Dissociative symptom severity, but not depression or trait anxiety scores, correlated with FA values within both identified sub-networks in the PTSD-D group. This further supports the assumption that these group differences are directly related to the dissociative symptomatology. In the first identified sub-network, patients with PTSD-D displayed significantly altered structural connectivity (i.e. lower FA values) between the left amygdala, hippocampus, and thalamus and higher FA between the left thalamus and the brain stem compared to patients with classic PTSD. The thalamus receives afferent sensory input from the brain stem via the internal capsule, while the fornix connects amygdala and hippocampus to the anterior nuclei of the thalamus (Catani et al. 2013). In healthy individuals, alterations in this limbic-thalamo circuit have been associated with altered consciousness (Blumenfeld 2012) and selective memory deficits (Carlesimo et al. 2011; Gilboa et al. 2006) – both phenomena observed in patients with PTSD-D. It has been suggested that during the traumatic event amygdala-mediated sensory representation of the scene is strengthened, disconnected from hippocampus-dependent contextual information, which gives rise to de-contextualized re-experiencing (Brewin et al. 2010). This modulation may be amplified if consciousness is lowered during dissociation. Congruently, peritraumatic dissociation has been identified as a strong predictor for intrusive symptomatology (Ozer et al. 2003), the severity of peritraumatic dissociation correlated with activation of brain structures subserving autobiographic memory recall (Daniels et al. 2012), and patients with PTSD-D display heightened intrusive symptom severity in some studies (D. J. Stein et al. 2013). Moreover, reduced amygdalar and hippocampal volume has been reported in women with dissociative identity disorder (DID) and comorbid PTSD, and dissociative symptom severity was found to be negatively correlated with hippocampal volume in women with PTSD due to childhood sexual abuse (Bremner et al. 2003; M. B. Stein et al. 1997). Interestingly, Felmingham et al. (2008) found heightened activity of the amygdala and parahippocampus in patients with PTSD-D only during the subliminal exposition of fearful faces. Thus, altered structural connectivity in limbic-thalamic circuits may present a pre-existing risk factor for sensory disintegration and an initial (pre-conscious) heightened limbic response to stress, leading to dissociation and exacerbation of integrative memory processes. Alternatively, the severity of trauma may modulate the emotional reaction and thus the likelihood that an individual is driven into an altered state of consciousness, regardless of the subject’s biological predisposition (cf. Lanius 2015; Putnam 1997). When this state is frequently re-activated as seen in PTSD-D, respective changes in the white matter microstructure may evolve. Our cross-sectional design limits weighting of either explanation. Yet, in both scenarios, it is conceivable that a dissociative response to a traumatic event and subsequent reminders may be adopted as a conscious coping style over time.

Our second exploratory results indicate altered structural connectivity between the left pallidum, left ventral DC, and left putamen. Our findings compliment previous work showing patients with PTSD and comorbid DID display larger bilateral putamen and right pallidum compared to PTSD-patients without DID (Chalavi et al. 2015). Chalavi et al. (2015) also found volumetric measurements of both structures to correlate positively with dissociative symptom severity. Activation of the head of the right caudate (adjacent to the putamen) has previously been associated with dissociative analgesia in PTSD (Mickleborough et al. 2011) and activation of the caudate with specific dissociative identity states (Reinders et al. 2014). The putamen (with the caudate part of the dorsal striatum) and the pallidum belong to the basal ganglia and are responsible for inhibiting and activating movement impulses, respectively. Excitatory and inhibitory direct pathways run between the pallidum, putamen, and the substantia nigra and subthalamic nuclei, respectively – both structures included in the ventral DC. It is possible that altered structural connectivity in these low-level motor-related structures underlie passive threat response such as freezing – a state that is assumed to be the homologue of dissociation in animals (for review see Hagenaars et al. 2014).

In conjunction, our explorative findings suggest that aberrations in subcortical inter-connectivity in PTSD-D is worth pursuing further. However, the results of our exploratory analysis ought to be replicated with pre-registration (Szucs and Ioannidis 2017). Once replicated in an assumption-testing study, we suggest that future studies should focus on contributing to a wider theoretical framework of altered subcortical rather than cortical processes in PTSD-D.

### Limitations

The generalization of our results is limited by the following factors: First, the findings presented here were not hypothesized a priori and thus need to be replicated in a confirmatory study. Second, to ensure ecological validity, we did not exclude patients with certain comorbidities or patients taking anti-depressants. However, only two patients took anti-depressant medication and we controlled for comorbid effects in our post-hoc analyses.

Third, our results cannot be generalized to men or women with traumatization during adulthood as our sample consisted exclusively of women with a history of childhood trauma. However, as the CTQ did not evidence a significant group difference with regard to the severity of childhood trauma, it seems unlikely that the observed group differences are related to the nature of the traumatic experience per se. Fourth, the results of the dissociation assessment tools employed for allocating participants into the two PTSD subgroups indicated that their selectivity is not absolute, as several participants of the classic PTSD group also exhibited a low level of dissociative symptoms. However, group allocation resulted in highly significant mean differences for all dissociation questionnaires, while keeping the two groups comparable with respect to all other assessed domains.

Fifth, the resolution of the data and FreeSurfer parcellation limits the interpretation; e.g. we cannot ascertain which specific subnuclei of the thalamus and the ventral DC are involved in the detected circuits. Sixth, general methodological issues apply in regard to the graph theoretical analysis of diffusion MRI tractography data. By using constrained spherical deconvolution tractography to reconstruct brain networks of white matter fiber bundles, which is capable of resolving crossing fiber tracts (Jeurissen et al. 2013), the number of false negative findings was decreased (J. D. Tournier et al. 2007). However, other challenges of the tracking algorithm, e.g. modelling distinctive fiber geometries, may increase false-positive streamlines and thus present a limitation. Finally, it should be considered that weighting the connectivity matrices with the diffusion parameter FA does not allow strong inferences of the state of the anatomical connection. Because FA is modulated by a variety of microstructural factors, lower or higher FA between regions does not present an implication for the degree of structural connectivity (D. K. Jones et al. 2013).

## Conclusion

The proposed model of over-regulation of limbic structures by prefrontal regions in PTSD-D is not underpinned by altered white matter connectivity on a network level and thus may rather present a dynamic neural process better detectable using functional neuroimaging. Our exploratory results however yielded interesting alterations in structural connectivity between subcortical areas in PTSD-D relative to classic PTSD, which suggest distinct low-level emotional, sensory, and motor processes that might give rise to dissociative responses during and after trauma.

Our findings may hold clinical implications by potentially supporting new avenues of interventions for patients with PTSD-D, in which emotion regulation strategies are strengthened before trauma-focussed therapy is implemented to treat intrusive symptomatology (cf. Cloitre et al. 2002; Steil et al. 2011). Respective therapeutic elements have already shown to effectively reduce dissociative symptoms in women with PTSD related to childhood abuse (Cloitre et al. 2012). Future longitudinal studies should investigate whether alterations in initial sensory encoding depict a risk factor to overregulate emotions and how this may inform advances for psychotherapeutic pre- and interventions for those affected.

## Electronic supplementary material

ESM 1 (DOCX 74 kb)

ESM 2 (DOCX 17 kb)

ESM 3 (DOCX 14 kb)

ESM 4 (DOCX 14 kb)

ESM 5 (DOCX 17 kb)
