# Supplementary material for: The dissociative subtype of posttraumatic stress disorder is associated with subcortical white matter network alterations
Source: Brain Imaging Behav. 2020 Apr 27;15(2):643–55. doi: 10.1007/s11682-020-00274-x (PMC8032639; doi:10.1007/s11682-020-00274-x)
Supplement: Supplementary file 1 — (DOCX 74 kb) [file 11682_2020_274_MOESM1_ESM.docx]

| **Online Resource 1 (Table)** | | | |
| --- | --- | --- | --- |
| Results of the partial correlation analysis (controlled for age) between dissociative symptom severity, as measured by the CDS-30, and interregional FA displayed for the PTSD-D and classic PTSD. At an initial-link threshold of *p_lt_*<.005, two subnetworks in the classic PTSD group and three sub-networks in the PTSD-D group were identified within which FA values correlated significantly with dissociative symptom severity. | | | |
| Sub-networks within FA correlated negatively with CDS-30 | | | |
| Classic PTSD (*n*=17) | *p_FWER_* | PTSD-D (*n*=22) | *p_FWER_* |
| (1) Left hippocampus **– –** Left caudate | .032 | (1) Right thalamus **+ +** Brain stem **+ +** Left hippocampus .027  **+ +**  **– –**  **– –**  Left thalamus Left amygdala | |
| (2) Right caudate **– –** Right thalamus | .032 | (2) Left putamen **+ +** Left ventral DC **+ +** Left pallidum | .029 |
|  |  | (3) Right precuneus **+ +** Left precuneus | .038 |
| CDS=Cambridge Depersonalization Scale; Lt=initial-link threshold; FA=fractional anisotropy; FWER=family wise error rate. Minus signs between brain regions (**– –**) represent connections for which FA correlated negatively with CDS-30 scores; plus signs between regions (**+ +**) represent connections for which FA correlated positively with CDS-30 scores. | | | |
