# Supplementary material for: The dissociative subtype of posttraumatic stress disorder is associated with subcortical white matter network alterations
Source: Brain Imaging Behav. 2020 Apr 27;15(2):643–55. doi: 10.1007/s11682-020-00274-x (PMC8032639; doi:10.1007/s11682-020-00274-x)
Supplement: Supplementary file 2 — (DOCX 17 kb) [file 11682_2020_274_MOESM2_ESM.docx]

| **Online Resource 2 (Table)**  Intercorrelations between questionnaire scores. | | | | | | | | | | | | |
| --- | --- | --- | --- | --- | --- | --- | --- | --- | --- | --- | --- | --- |
| Questionnaire | BDI | CAPS | CDS_30 | CTQ sum | DES | ERQ-R | ERQ-S | MDI | PCL | PDEQ | SQR-20 | STAI-T |
| BDI | 1 |  |  |  |  |  |  |  |  |  |  |  |
| CAPS | .24 | 1 |  |  |  |  |  |  |  |  |  |  |
| CDS_30 | .32* | .38* | 1 |  |  |  |  |  |  |  |  |  |
| CTQ sum | .17 | .07 | .22 | 1 |  |  |  |  |  |  |  |  |
| DES | .12 | -.07 | -.09 | -.13 | 1 |  |  |  |  |  |  |  |
| ERQ-R | .33* | .33* | .15 | .01 | -.21 | 1 |  |  |  |  |  |  |
| ERQ-S | -.21 | -.34* | -.39* | -.07 | -.10 | .04 | 1 |  |  |  |  |  |
| MDI | .36* | .44** | .81*** | .16 | -.10 | .22 | -.25 | 1 |  |  |  |  |
| PCL | .23 | .27 | .57*** | .27 | .04 | .07 | -.11 | .55*** | 1 |  |  |  |
| PDEQ | -.08 | .40* | .57*** | .25 | -.17 | .003 | -.12 | .48** | .51** | 1 |  |  |
| SDQ-20 | .37* | .36* | .81*** | .37* | -.08 | .28 | -.18 | .83*** | .54*** | .57*** | 1 |  |
| STAI-T | .63** | .45** | .51** | .10 | -.13 | .49** | -.24 | .42** | .43** | .15 | .40* | 1 |
| BDI=Beck Depression Inventory; CAPS=Clinician-Administered PTSD Scale; CDS=Cambridge Depersonalization Scale; CTQ=Childhood Trauma Questionnaire; DES=Dissociative Experiences Scale; ERQ-R=Emotion Regulation Questionnaire Reappraisal; ERQ-S=Emotion Regulation Questionnaire Suppression; MDI=Multiscale Dissociation Inventory; PDEQ=Peritraumatic Dissociative Experiences Questionnaire; SDQ=Somatoform Dissociation Questionnaire; STAI-T=State-Trait Anxiety Scale, trait version;  *Correlation is significant at *p*<.05 (2-tailed)  **Correlation is significant at *p*<.01 (2-tailed)  ***Correlation is significant at *p*<.001 (2-tailed) | | | | | | | | | | | | |
