# Supplementary material for: The dissociative subtype of posttraumatic stress disorder is associated with subcortical white matter network alterations
Source: Brain Imaging Behav. 2020 Apr 27;15(2):643–55. doi: 10.1007/s11682-020-00274-x (PMC8032639; doi:10.1007/s11682-020-00274-x)
Supplement: Supplementary file 3 — (DOCX 14 kb) [file 11682_2020_274_MOESM3_ESM.docx]

| **Online Resource 3 (Table)**  Results of the partial correlation analysis (controlled for age) between trait anxiety, as measure by the STAI-T, and interregional FA in the PTSD-D group only. At an applied initial-link threshold of *p_lt_*<.005, three sub-networks were identified within FA values correlated with STAI-T scores. | |
| --- | --- |
| Sub-networks within FA correlated with STAI-T scores | *p_FWER_* |
| Right rostral middle frontal gyrus **– –** Left rostral middle frontal gyrus | .040 |
| Right ventral diencephalon **– –** Right putamen | .040 |
| Right precuneus **+ +** Left precuneus | .040 |
| Right caudate **+ +** Right thalamus | .040 |
| Lt=initial-link threshold; PTSD-D=dissociative subtype of posttraumatic stress disorder; FA=fractional anisotropy; FWER=family wise error rate, STAI-T=State-Trait Anxiety Scale, trait version; Minus signs between brain regions (**– –**) represent connections for which FA correlated negatively with STAI-T scores; plus signs between regions (**+ +**) represent connections for which FA correlated positively with STAI-T scores. | |
