# Supplementary material for: The dissociative subtype of posttraumatic stress disorder is associated with subcortical white matter network alterations
Source: Brain Imaging Behav. 2020 Apr 27;15(2):643–55. doi: 10.1007/s11682-020-00274-x (PMC8032639; doi:10.1007/s11682-020-00274-x)
Supplement: Supplementary file 4 — (DOCX 14 kb) [file 11682_2020_274_MOESM4_ESM.docx]

| **Online Resource 4 (Table)**  Results of the partial correlation analysis (controlled for age) between depressive symptoms, as measure by the BDI-II, and interregional FA in the PTSD-D group only. At an applied initial-link threshold of *p_lt_*<.005, four sub-networks were identified within FA values correlated with BDI-II scores. | |
| --- | --- |
| Sub-networks within FA correlated with BDI-II scores | *p_FWER_* |
| Right rostral middle frontal gyrus **– –** Left rostral middle frontal gyrus | .042 |
| Right ventral diencephalon **– –** Right putamen | .042 |
| Braim stem **– –** Left caudate | .042 |
| Right precuneus **+ +** Left precuneus | .042 |
|  |  |
| Lt=initial-link threshold; PTSD-D=dissociative subtype of posttraumatic stress disorder; FA=fractional anisotropy; FWER=family wise error rate, BDI-II=Beck Depression Inventory; Minus signs between brain regions (**– –**) represent connections for which FA correlated negatively with BDI-II scores; plus signs between regions (**+ +**) represent connections for which FA correlated positively with BDI-II scores. | |
