# Supplementary material for: The dissociative subtype of posttraumatic stress disorder is associated with subcortical white matter network alterations
Source: Brain Imaging Behav. 2020 Apr 27;15(2):643–55. doi: 10.1007/s11682-020-00274-x (PMC8032639; doi:10.1007/s11682-020-00274-x)
Supplement: Supplementary file 5 — (DOCX 17 kb) [file 11682_2020_274_MOESM5_ESM.docx]

| **Online Resource 5 (Table)** | |
| --- | --- |
| Results of the group comparison (controlled for age) after excluding patients with secondary borderline personality disorder (*n*=6). At an initial-link threshold of *p_lt_*<.005, two subnetworks were identified for which patients with PTSD-D displayed altered FA compared to patients with classic PTSD. | |
| Significant subnetworks | *p_FWER_* |
| (1) Left amygdala **– –** Left hippocampus **– –** Left thalamus **+ +** Brain stem  **– –**  Left caudate | .024 |
| (2) Left putamen **+ +** Left ventral diencephalon **+ +** Left pallidum | .031 |
| Lt=initial-link threshold; FA=fractional anisotropy; FWER=family wise error rate. Minus signs between brain regions (**– –**) represent connections, for which patients with PTSD-D displayed lower FA than patients with classic PTSD; plus signs between regions (**+ +**) represent connections, for which the PTSD-D group displayed lower FA than the classic PTSD group. | |
